# Low-density lipoprotein cholesterol goal attainment in Germany: Results from the DA VINCI study

**DOI:** 10.1016/j.athplu.2022.07.024

**Published:** 2022-08-08

**Authors:** Ioanna Gouni-Berthold, Frank Schaper, Ulrike Schatz, Anja Tabbert-Zitzler, Uwe Fraass, Sarah Sauer, Kausik K. Ray

**Affiliations:** a University of Cologne, Center for Endocrinology, Diabetes and Preventive Medicine, University of Cologne, Faculty of Medicine and University Hospital, Cologne, Germany; b Diabetology Clinic, Dresden, Germany; c University Hospital “Carl Gustav Carus”, Technische Universität, Dresden, Germany; d Amgen Germany GmbH, Munich, Germany; e Imperial Centre for Cardiovascular Disease Prevention and Imperial Clinical Trials Unit, Imperial College, London, UK

**Keywords:** Guidelines, Cholesterol, Statins, Dyslipidaemia, Cardiovascular risk, Da Vinci, Goal attainment

## Abstract

**Background and aims:**

Cardiovascular mortality is high in Germany. For patients with high or very high cardiovascular risk, the European Society of Cardiology (ESC)/European Atherosclerosis Society (EAS) guidelines recommend intensive lipid lowering therapy (LLT). This study aimed to assess dyslipidaemia management and achievement of the ESC/EAS guideline-recommended low-density lipoprotein cholesterol (LDL-C) goals.

**Methods:**

This European 18-country, cross-sectional, observational DA VINCI study (EUPAS22075) collected data during a single visit between June 2017 and November 2018 and included LLT in the preceding 12 months and the patients' most recent LDL-C measurement. Achievement of the risk-based 2016 and 2019 ESC/EAS LDL-C goals while receiving stabilized LLT was assessed. Data from the German cohort are presented here.

**Results:**

Seven German sites enrolled a total of 421 primary and secondary prevention patients, 327 were receiving stabilized LLT at the time of LDL-C measurement, i.e. statin monotherapy of high (16%; n = 53), moderate (49%; n = 160) or low (7%; n = 24) intensity, ezetimibe combination (18%; n = 58), proprotein convertase subtilisin/kexin type 9 antibody combination (3%; n = 9), and other LLT (7%; n = 23). The 2016 and 2019 risk-based LDL-C goals were attained by 46% (n = 149) and 28% (n = 92) of patients, respectively.

**Conclusions:**

There is a large gap between ESC/EAS recommendations and LDL-C goal achievement in routine clinical practice in high and very high-risk patients in Germany. Low-to-moderate-intensity statin monotherapy was the most frequently used LLT; use of high-intensity statins and combination therapy was limited. In addition to optimizing statin intensity, combination with non-statin LLT may be needed in most of these patients.

## Highlights

• This study evaluated lipid-lowering therapy use and dyslipidemia burden in Germany.
• Attainment of LDL-C goals set by the 2016 and 2019 EAS/ESC guidelines was evaluated.
• In Germany, more than half of patients did not achieve their 2016 LDL-C goals.
• Attainment of the more stringent 2019 LDL-C goals was <30%.
• Intense combination therapy in Germany could lead to better LDL-C goal attainment.

## Graphical abstract

**Figure:**
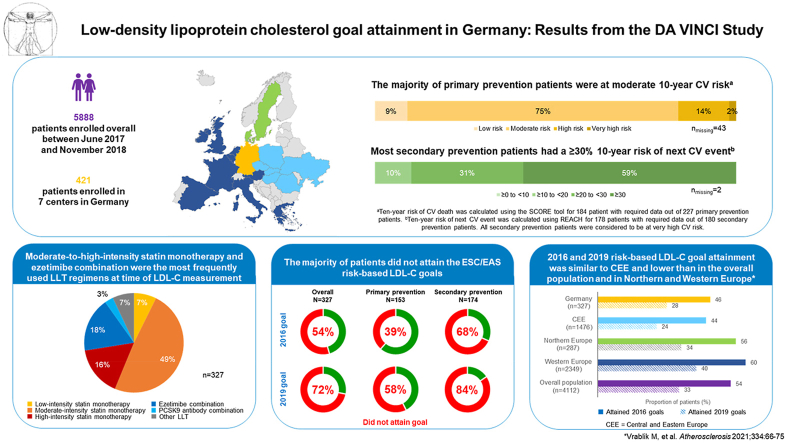

## Introduction

As in most industrialized countries [1,2], cardiovascular (CV) events are the most frequent cause of death in Germany [3]. Plasma low-density lipoprotein cholesterol (LDL-C) levels are causally linked to atherosclerosis and lowering LDL-C has been shown to improve clinical outcomes [[4], [5], [6]]. There is no known lower LDL-C threshold at which the degree of risk reduction is attenuated [7,8]. The European Society of Cardiology (ESC) and the European Atherosclerosis Society (EAS) have recommended LDL-C goals for patients of all CV risk levels; recommended LDL-C goals became more stringent in the 2019 [9] compared with the 2016 [10] edition. For high-risk patients, a ≥50% reduction from untreated (or extrapolated baseline if treated) LDL-C levels OR attainment of the recommended goal of LDL-C <100 mg/dL (<2.6 mmol/L) in 2016 was updated to a ≥50% reduction AND attainment of the stricter goal of LDL-C <70 mg/dL (<1.8 mmol/L). Likewise, for very-high risk patients, 2016 recommendations of a ≥50% LDL-C reduction from baseline OR attainment of the recommended LDL-C goal of <70 mg/dL (<1.8 mmol/L), were updated in 2019 to a ≥50% reduction AND attainment of LDL-C <55 mg/dL (<1.4 mmol/L). A new category of patients who had experienced a second CV event within two years was also introduced in 2019, for whom a ≥50% reduction of LDL-C levels and attainment of LDL-C <40 mg/dL (<1.0 mmol/L) was recommended. European and global studies such as EUROASPIRE V [[11], [12], [13]] and DYSIS II [14,15] and also recent studies from Germany [[16], [17], [18]] have shown that attainment of guideline-recommended goals is difficult to achieve in clinical practice. Statins alone may be insufficient to control LDL-C in many patients and combination therapies may be needed to lower LDL-C levels to and below the recommended thresholds [19] and thus decrease the risk of CV events.

The aim of the pan-European DA VINCI study [[20], [21], [22]] was to assess how current clinical practice of dyslipidaemia treatment impacts LDL-C goal attainment. DA VINCI included patients treated in both primary and secondary care, allowing the description of treatment patterns in diverse healthcare settings. Here we present the data from the pre-specified sub-group analysis of the German cohort.

## Patients and methods

DA VINCI was a pan-European cross-sectional study enrolling adults (≥18 years) receiving lipid-lowering therapy (LLT) for primary or secondary prevention at primary and secondary care clinics across Europe. In primary care centers, care was provided by community physicians or general practitioners, while in secondary care, it was provided by hospital specialists. Primary and secondary prevention patients were managed in both primary and secondary care settings. For patients in primary prevention at LDL-C measurement date, 10-year CV risk was estimated using systematic coronary risk evaluation (SCORE) [23]. Patients were subsequently categorized as low–moderate, high, or very high risk according to the ESC/EAS guidelines, where low risk = 0, moderate risk = 1–4, high risk = 5–9, and very high risk = ≥10. Primary prevention enrolled on the basis of high-risk conditions such as diabetes, familial hypercholesterolaemia, and reduced glomerular filtration rate were categorized as per ESC/EAS risk categories (low, moderate, high, very high). All secondary prevention patients were classified as very-high risk and included those with established atherosclerotic cardiovascular disease (ASCVD), such as peripheral artery disease, ischemic stroke, or coronary disease. Additionally, 10-year risk of fatal and non-fatal cardiovascular events (REACH) [24] was calculated for secondary prevention patients.

Data were collected from patients' medical records (Fig. S1) including patient demographics and clinical characteristics and relevant past medical history (CV events including dates, known risk factors [smoking, family history], diabetic/hypertension/renal status, vascular bed involvement). The patients' most recent LDL-C value recorded within the 14 months prior to (and including) the enrolment visit, LLT at the enrolment visit and in the preceding 12 months, history of intolerance to any statin at any dose, the reason for LLT prescription in patients without previous ASCVD events, and concomitant medications, were also collected. The enrolment period of the DA VINCI study was between June 21, 2017 and November 20, 2018. The study methods are described in more detail in the primary DA VINCI publication [20].

### Aims and outcomes

The primary study outcome was the percentage of patients achieving the LDL-C goals recommended in the 2016 ESC/EAS dyslipidaemia guidelines while receiving stabilized LLT (defined as no change in LLT dose or regimen for at least 28 days before the LDL-C measurement date). The secondary outcome was LLT use (type, dose, frequency; including combination therapy), as assessed at the enrolment date. An exploratory post-hoc analysis of the percentage of patients achieving the LDL-C goals recommended in the 2019 ESC/EAS guidelines, was also performed.

### Statistical analysis

All analyses were descriptive. Continuous variables were reported as mean and standard deviation (SD) or standard error (SE) for normally distributed data, and as median and 25th and 75th percentiles (Q1 and Q3, respectively) for data with a skewed distribution. For categorical variables, the number and percentage of patients in each category were reported.

### Ethics declaration

The study protocol was approved by the institutional review board or independent ethics committee at each participating site (full protocol available online [ENCePP; registration no. EU PAS 22075]). All enrolled persons gave their informed consent prior to their inclusion in the study. This study has thus been performed in accordance with the 1975 Declaration of Helsinki.

## Results

### Study population

The German study cohort included 421 patients, enrolled at seven sites (three primary care [43%] and four secondary care [57%] sites). The German cohort thus represents 7% of all 5888 patients participating in the DA VINCI study. Most patients were male (64%, n = 269) and Caucasian (96%, n = 404). The mean age was 67 (SD 12) years (Table 1, Fig. S2). Overall, 53% (n = 225) of patients were in primary prevention and 47% (n = 196) were in secondary prevention. Two patients counted as secondary prevention patients at the enrolment visit were primary prevention patients at the LDL-C measurement date but had a cardiovascular event between LDL-C measurement date and the enrolment visit. Therefore, there were 227 primary prevention patients at the LDL-C measurement date. Fifty-two percent (n = 219) were ever-smokers and 55% (n = 231) had been diagnosed with type 2 diabetes mellitus. Compared with the overall European population, German patients were slightly older (67 versus 65 years) and there was a larger number of patients with diabetes mellitus type 2 (55% versus 36%).

**Table 1:** Patient demographics and clinical characteristics at enrolment for the German cohort and the DA VINCI study overall.

	Germany N = 421	Overall [20] N = 5888
Male, n (%)	269 (63.9)	3413 (58.0)
Ethnicity, white, n (%)	404 (96.0)	5435 (92.3)
Age (years), mean (SD)	67.4 (12.1)	65.1 (11.9)
Systolic blood pressure (mmHg), mean (SD)	136.4 (17.5)	134.8 (17.1)
Diastolic blood pressure (mmHg), mean (SD)	80.8 (10.5)	78.0 (10.7)
BMI (kg/m^2^), median (Q1, Q3)	29.2 (25.8, 32.2)	28.7 (25.1, 31.6)
Smoking history, n (%)		
Non-smoker	202 (48.0)	2854 (48.5)
Ex-smoker	158 (37.5)	2059 (35.0)
Light smoker	17 (4.0)	313 (5.3)
Moderate smoker	30 (7.1)	391 (6.6)
Heavy smoker	14 (3.3)	253 (4.3)
Missing	0 (0)	18 (0.3)
Diabetes mellitus, type 2, n (%)	231 (54.9)	2112 (35.9)
Chronic kidney disease ≥ grade 3, n (%)	81 (19.2)	432 (7.3)
Familial hypercholesterolemia, n (%)	24 (5.7)	284 (4.8)
Vascular bed involvement, n (%) [a]		
Coronary	82 (19.5)	1007 (17.1)
Cerebrovascular	72 (17.1)	1296 (22.0)
Peripheral	82 (19.5)	1125 (19.1)

ASCVD = atherosclerotic cardiovascular disease; SD = standard deviation.

^a^ More than one vascular bed can be involved in a given patient; the vascular bed primarily being managed at enrolment is shown as type of established ASCVD in Fig. S2.

### Cardiovascular risk profile

SCORE or REACH at the time of LDL-C measurement could not be calculated for 43 and 2 patients, respectively, because of missing data. SCORE was thus calculated for the subset of 184 out of 227 patients in the primary prevention group. REACH was calculated for the subgroup of 178 out of 180 patients with established ASCVD (secondary prevention group). Most primary prevention patients as determined by SCORE (75%; n = 138) had a moderate 10-year risk of fatal cardiovascular disease; the median risk by SCORE was 2.0 (Q1 1.0, Q3 4.0). Most secondary prevention patients (59%; n = 105) had ≥30% 10-year risk of another CV event as determined by REACH, with 31% (n = 55) having ≥20 to <30% CV risk; the median risk by REACH was 33.1% (Q1 25.0, Q3 43.5). Compared to the overall population, in Germany more primary prevention patients had moderate risk (75% versus 69%) while fewer patients had high risk (14% versus 19%). The secondary prevention patients had comparable risk distribution (Fig. S3).

### Lipid lowering therapy

**Fig. 1:**
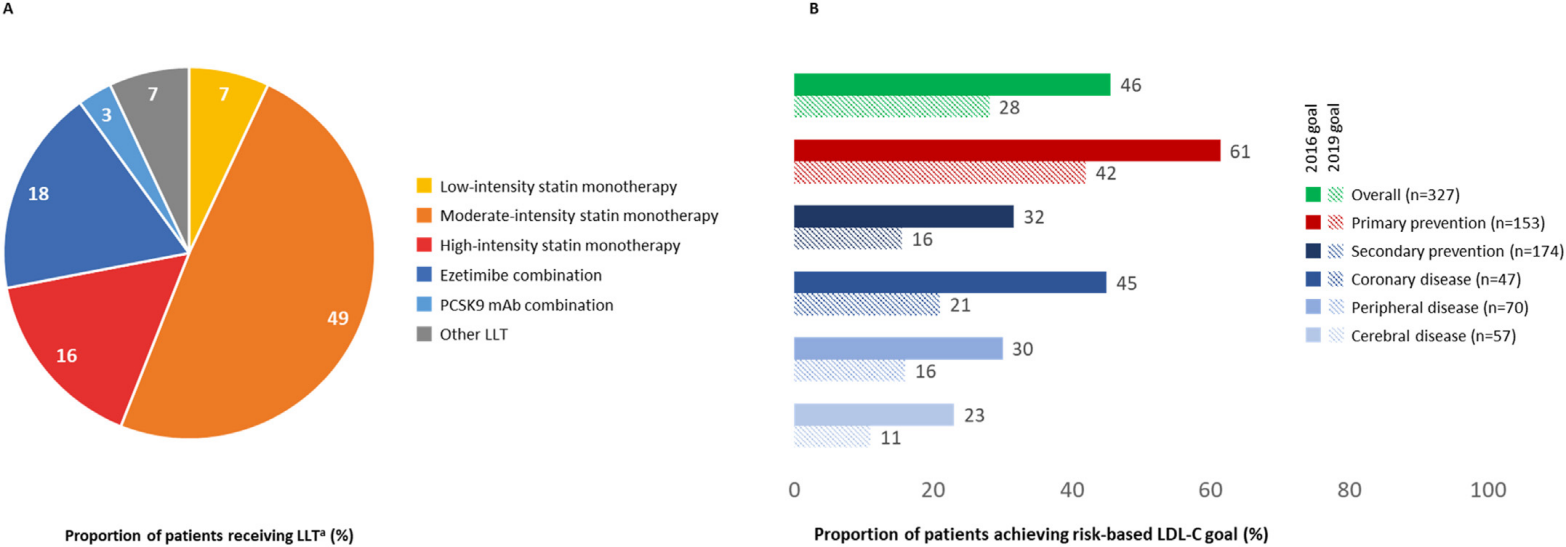
Stabilized lipid-lowering therapies (A) and ESC/EAS goal attainment (B) in Germany ASCVD = atherosclerotic cardiovascular disease; EAS = European Atherosclerosis Society; ESC = European Society of Cardiology; LDL-C = low-density lipoprotein cholesterol; LLT = lipid-lowering therapy; PCSK9 mAb = proprotein convertase subtilisin/kexin type 9 monoclonal antibody. ^a^ corresponds to “All LLT – stabilized LLT” in the German cohort in Table 2. Stabilized LLT was defined as no change in LLT dose or regimen for at least 28 days prior to LDL-C measurement. Patients who were in secondary prevention at the visit date, but whose first ASCVD event occurred after the date of their stabilized LDL-C were categorized as primary prevention patients. 2016/2019 risk-based LDL-C goals used for analysis [9,10]: •Low risk: 2016/2019, <116 mg/dL •Moderate risk: 2016, <116 mg/dL; 2019, <100 mg/dL •High risk: 2016, <100 mg/dL; 2019, <70 mg/dL •Very high risk: 2016, <70 mg/dL; 2019, <55 mg/dL.

Of the patients stabilized on their respective LLT (n = 327) and evaluable for goal attainment, most patients received moderate-intensity statin monotherapy (49%; n = 160), with 16% (n = 53) receiving high-intensity statin monotherapy, and 7% (n = 24) receiving low-intensity statin monotherapy. Ezetimibe was administered in combination with moderate to high-intensity statins in 18% (n = 58) of patients, PCSK9 antibody combination therapy was administered to 3% (n = 9; Fig. 1A). Compared with the overall study population, patients in Germany received high-intensity statin monotherapy less frequently (16% versus 28%), but more patients received ezetimibe combination therapy (18% versus 9%) or PCSK9 antibody combination therapy (3% versus 1%; Table 2).

**Table 2:** Use of lipid-lowering therapy.

Lipid-lowering therapy, n (%)	LLT at enrolment^a^		Stabilized LLT^b^	
	Germany N = 421	Overall [20] N = 5888	Germany N = 327	Overall [20] N = 4112
**Any LLT**^c^				
Any statin	394 (93.6)	5554 (94.3)	305 (93.3)	3856 (93.8)
High-intensity statin	89 (21.1)	2028 (34.4)	72 (22.0)	1306 (31.8)
Moderate-intensity statin	239 (56.8)	3164 (53.7)	177 (54.1)	2279 (55.4)
Low-intensity statin	34 (8.1)	226 (3.8)	28 (8.6)	171 (4.2)
Unknown intensity statin	32 (7.6)	136 (2.3)	28 (8.6)	100 (2.4)
Ezetimibe	87 (20.7)	667 (11.3)	78 (23.9)	491 (11.9)
PCSK9 antibody	14 (3.3)	81 (1.4)	12 (3.7)	59 (1.4)
Fibrates	16 (3.8)	248 (4.2)	15 (4.6)	181 (4.4)
Fish oils	23 (5.5)	43 (0.7)	20 (6.1)	36 (0.9)
Other	3 (0.7)	16 (0.3)	3 (0.9)	12 (0.3)
**All LLT**^d^				
Statin monotherapy				
High-intensity statin monotherapy	69 (16.4)	1787 (30.3)	53 (16.2)	1134 (27.6)
Moderate-intensity statin monotherapy	220 (52.3)	2966 (50.4)	160 (48.9)	2131 (51.8)
Low-intensity statin monotherapy	29 (6.9)	194 (3.3)	24 (7.3)	148 (3.6)
Ezetimibe combination	65 (15.4)	516 (8.8)	58 (17.7)	380 (9.2)
PCSK9 antibody combination	10 (2.4)	64 (1.1)	9 (2.8)	49 (1.2)
Other	28 (6.7)	361 (6.1)	23 (7.0)	270 (6.6)

LLT = lipid-lowering therapy; PCSK9 = proprotein convertase subtilisin/kexin type 9.

Fibrates: bezafibrate, clofibrate, ciprofibrate, clofibride, clinofibrate, gemfibrozil, etofibrate, fenofibrate, ronifibrate, simfibrate.

Statin intensity was defined per the American College of Cardiology/American Heart Association definition [32].

a Use of LLT continuing at enrolment.

b Use of LLT at the time of LDL-C measurement where LLT is stabilized (no change in LLT dose or regimen for at least 28 days prior to the LDL-C measurement date) for those patients evaluable for goal attainment.

c Any use of a specific LLT regardless of whether a patient also received any other LLT.

d All LLT used by each patient.

### LDL-C levels and ESC/EAS goal attainment

**Fig. 2:**
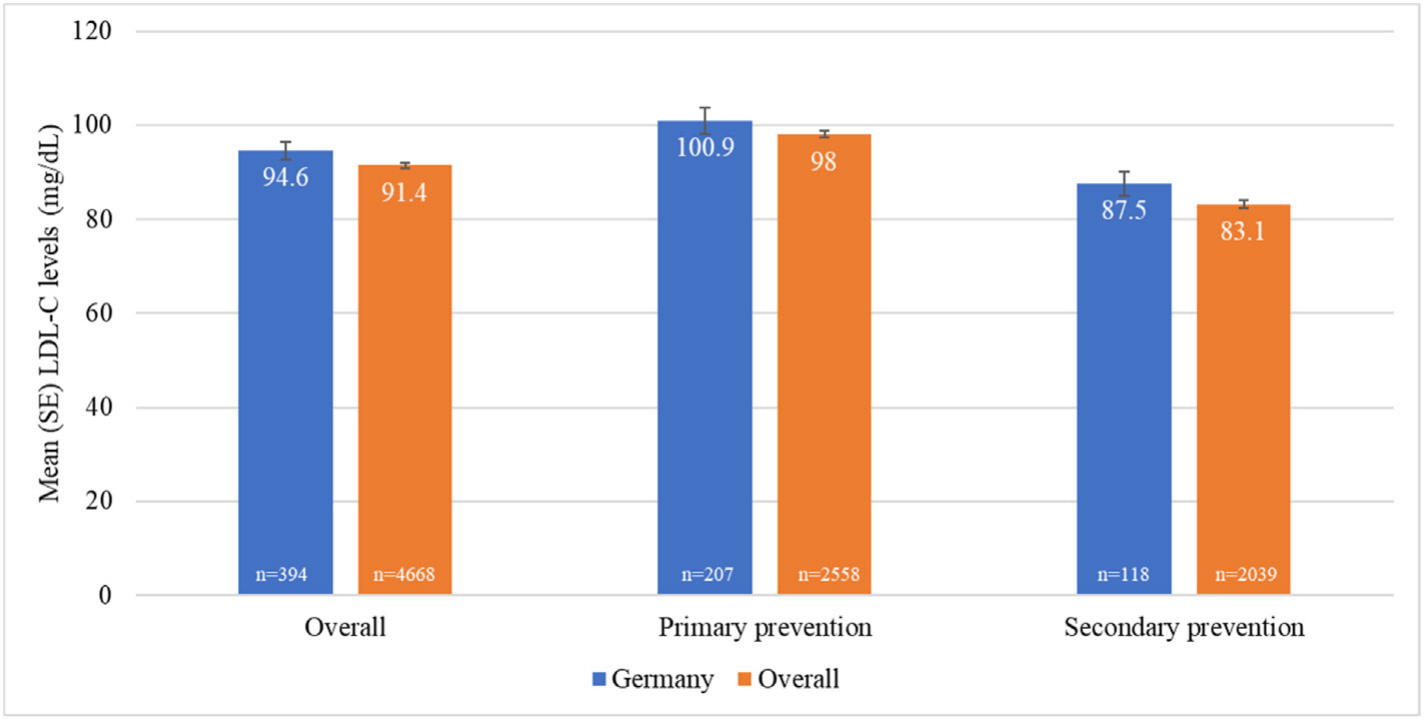
**Mean low-density lipoprotein cholesterol levels in patients on stabilized lipid-lowering therapy**^a^ LDL-C = low-density lipoprotein-cholesterol. n is the number of patients in each category on stabilized lipid-lowering therapy and with non-missing low-density lipoprotein cholesterol goal data. ^a^ No change in lipid-lowering therapy dose or regimen for at least 28 days prior to LDL-C measurement.

Patients on stabilized LLT had a mean LDL-C level of 94.6 (SE 1.9) mg/dL, with slightly higher LDL-C levels observed in primary versus secondary prevention patients (Fig. 2). Overall, the risk-based LDL-C goals recommended by the 2016 ESC/EAS guidelines were attained by 46% (n = 149) of patients; the 2019 LDL-C goals were attained by 28% (n = 92) (Fig. 1B). Among patients with different types of established ASCVD, achievement of the 2016 LDL-C goal of <70 mg/dL for very high-risk patients ranged from 23% to 45%. Attainment of LDL-C levels <55 mg/dL, as recommended for very high-risk patients in the 2019 guidelines, ranged from 11% to 21%. Compared with the overall study population, the mean LDL-C level was slightly higher (94.6 mg/dL versus 91.4 mg/dL) and LDL-C goal attainment was lower in Germany (2016 goals: 46% versus 54%, 2019 goals: 28% versus 33%, respectively).

## Discussion

The present analysis from the DA VINCI study estimated the achievement of the LDL-C goals recommended by the 2016 ESC/EAS guidelines [10] in routine clinical practice in Germany. In addition, a post hoc analysis explored attainment of the 2019 ESC/EAS LDL-C goals [9] – the first of its kind in Germany. Overall, attainment of the risk-based LDL-C goals was suboptimal with 46% of patients attaining the 2016 goal and 28% achieving the 2019 goal. Interestingly, LDL-C goal attainment was lower than in the overall DA VINCI study population as reported by Ray and colleagues [20], with 54% and 33%, respectively. This trend was consistent for the primary prevention setting (53% of the German cohort, mostly moderate CV risk), where risk-based goal attainment was 61% in the current analysis versus 68% in the study as a whole [20] for the 2016 LDL-C goals, and 43% versus 48% [20], respectively, for the 2019 goals. For secondary prevention (43% of German patients, all considered very high-risk), 2016 LDL-C goal attainment was 32% in the German analysis versus 39% in the study as a whole [20] and 16% versus 19% [20], respectively, for the 2019 goals.

The baseline characteristics of patients treated in Germany were generally comparable to the overall DA VINCI study population. Nevertheless, important CV risk factors such as type 2 diabetes mellitus and chronic kidney disease were numerically more frequent in the German cohort. The observed higher prevalence may result from differences in recruiting site characteristics rather than differences in the German CVD population as a whole. LDL-C levels were also slightly higher in the German analysis (mean 94.6 mg/dL) compared with the pooled overall population (mean 91.4 mg/dL [20]). The 10-year CV risk in patients in Germany was slightly lower in primary prevention with more moderate risk and fewer high-risk patients compared to the overall population, while in secondary prevention, CV risk was similar to the overall population. Dyslipidaemia management, however, differed from the overall data with less frequent use of high-intensity statin in Germany (16% compared to 28% overall). Use of combination ezetimibe plus statin (18%) or PCSK9 antibody plus statin (3%) therapy was more frequent in Germany compared with the overall population (9% and 1%, respectively), but was still at a low level overall. It should be noted that the overall DA VINCI population is a pooled dataset comprising data from 18 countries, where the pooled data differs from the individual country results, as exemplified by the LDL-C goal attainment by country shown in the overall data publication [20], as well as more detailed reports from the Austrian [21] cohort and Central and Eastern European countries [22].

In Germany, dyslipidaemia management practices and LDL-C goal attainment have been widely studied over the years in different datasets and from various perspectives, such as general practice of dyslipidaemia management, cardiac rehabilitation, or familial hypercholesterolaemia. Germany participated in the Dyslipidaemia International Study (DYSIS) I [25] and DYSIS II [14,15,26], and has extensive data from the post-MI cardiac rehabilitation-focussed PATIENT CARE registry [16], the familial hypercholesterolemia CaRe High registry [17], and other large datasets [18,27]. Even though these studies included very different patient populations, there was a consistent finding that LDL-C goal attainment is suboptimal, irrespective of the LDL-C goals recommended at the time by the ESC/EAS guidelines. This has most notably been observed in EUROASPIRE V [11,12]. Importantly, DYSIS II showed that suboptimal lipid management and the associated low LDL-C goal attainment was not corrected even when patients were hospitalized for an acute coronary event [26]. The consistent finding of suboptimal dyslipidaemia management is especially important in light of the 2019 iteration of the ESC/EAS guidelines [9] that recommend more stringent LDL-C goals to accommodate an increasing body of evidence showing that further lowering of LDL-C levels reduces CV risk [[4], [5], [6]].

However, although the majority of specialist guidelines follow the recommendations of the ESC/EAS, a small number of German national recommendations deviate therefrom. For primary prevention in the general practice setting [28] the German society of general and family medicine (Deutsche Gesellschaft für Allgemeinmedizin und Familienmedizin; DEGAM) issued guidelines in 2017, which were valid until the end of 2021. The DEGAM guidelines [28] recommend the use of fixed dose statins decoupled from any LDL-C goals. They describe, however, the possibility of dosing statins in order to achieve risk-based LDL-C goals based on SCORE, aiming at LDL-C <70 mg/dL for patients with SCORE >10%, LDL-C <100 mg/dL for patients with SCORE between 5% and 10%, and LDL-C level of <115 mg/dL when SCORE is between 1% and 5%. DEGAM discourages the use of high-intensity statins in patients without manifest coronary heart disease [28]. For patients with established ASCVD national care guidelines [29] (Nationale Versorgungsrichtlinie [NVL], valid from April 2019 to March 2024) exist. The NVL recommend the use of statins as primary LLT and discusses both, a fixed-dose strategy and an LDL-C goal-based strategy with an LDL-C goal of <70 mg/dL and additionally a reduction of >50% for patients with a baseline LDL-C of 70–135 mg/dL. If these goals are not achieved at maximally tolerated statin dose, ezetimibe should be added and if LDL-C remains above 140 mg/dL PCSK9 inhibitor can be offered [29]. These recommendations are less stringent than the recommendations of the ESC/EAS and are highly debated among specialists. Nevertheless they may explain, why more than half of patients participating in the DA VINCI German cohort did not attain the 2016 ESC/EAS goals.

All of the described studies of German clinical practice demonstrate that optimal LDL-C levels are often unattainable using monotherapy based approaches and that LLT regimens need to be intensified in many patients using medicines such as PCSK9 antibodies and other novel compounds with established positive cardiovascular outcomes [19,30,31]. Supporting data come from the overall DA VINCI dataset [20], which included enough patients to allow for subgroup analyses of LDL-C goal attainment by treatment intensity. In the overall study population, goal attainment was found to increase with LLT intensity, especially in the very high-risk population overall, and in the secondary prevention setting. With respect to the 2016 LDL-C recommendations, goal attainment was 20% in very high-risk patients overall and 19% for very high-risk patients on low-intensity statins, whereas 45% of patients in either setting receiving high-intensity statins attained their goal; 53% of very high-risk and 54% of secondary prevention patients on ezetimibe combination therapy and 67% of patients in either setting receiving PCSK9 antibody combination therapy attained the 2016 LDL-C goal [20].

Various limitations of the DA VINCI study design do, however, need to be considered. DA VINCI was a cross-sectional study with no longitudinal follow-up. There might be a degree of selection bias based on the different recruiting site characteristics in each country, i.e. primary or secondary care centers, different specialties such as cardiologists, endocrinologists, rehabilitation centers, etc. In some countries, prescriptions of combination therapies including PCSK9 antibodies are limited to certain specialists, e.g. endocrinologists. Additionally, the LDL-C threshold for initiation of combination therapies including PCSK9 antibodies varies depending on each country's reimbursement rules. Therefore, comparisons between the German cohort and the overall study population remain purely descriptive and are meant to provide context only. However, the main conclusion that there is a gap between the guidelines and clinical practice was confirmed in all countries. The degree to which LDL-C goals are attained in an individual patient depends – among other factors – on their pre-treatment LDL-C level. However, baseline/untreated LDL-C levels were unavailable in the DA VINCI study, therefore, it was not possible to quantify the proportion of patients achieving the exploratory ESC/EAS goal of a ≥50% LDL-C reduction from untreated levels. Goal attainment is also dependent on the choice of LLT, which is subject to physician bias and local variability in reimbursement. It was not possible to analyse goal attainment by LLT type in the present study, due to the small sample sizes for some subgroups. DA VINCI was conducted while the 2016 iteration of the ESC/EAS dyslipidaemia guidelines was used. The exploratory analysis conducted to estimate LDL-C goals recommended in the 2019 iteration was conducted post hoc. These newer goals therefore do not reflect treatment goals aimed at in routine practice at the time of study conduct. Finally, patients in Germany were enrolled across seven sites, which may not be representative of all German patients treated in clinical practice. At the site level, however, all eligible patients were invited in chronological order until the local enrolment target had been achieved to avoid enrolment bias based on conscious or unconscious investigator preference.

## Conclusions

There is a large gap between ESC/EAS 2016 dyslipidaemia management recommendations and LDL-C goal achievement in routine clinical practice in high- and very high-risk ASCVD patients in Germany, in line with findings from the other European countries participating in the DA VINCI study. This discrepancy is even more pronounced when the findings are projected to the more stringent LDL-C goals recommended in the 2019 ESC/EAS guidelines, which were published, however, after the completion of the DA VINCI study. Low-to-moderate intensity statin monotherapy remained the most frequently used LLT, while the use of high-intensity statins or combination therapy was limited. In addition to optimizing statin intensity, combining this with non-statin LLT, such as ezetimibe and a PCSK9 antibody, will be needed for many patients. In particular, achievement of the 2019 ESC/EAS LDL-C goal for high- and very high-risk patients appears to be largely unattainable on high-intensity statin monotherapy. Findings from the DA VINCI study suggest that around four in five very high-risk patients will likely require triple combination therapy including medications, preferably with established positive cardiovascular outcomes, such as a statin, ezetimibe and PCSK9 antibodies.

## Financial support

This analysis was funded by Amgen (Europe) GmbH.

## Author contributions

IGB, FS, and US were involved in patient enrolment and data acquisition for the German study cohort. KKR contributed to the development of the study protocol and to patient enrolment and data acquisition for the overall study population. All authors substantially contributed to the interpretation of the study results. All authors were involved in drafting of the manuscript, provided critical revisions for important intellectual content, approved the final version submitted for publication, and agreed to be accountable for all aspects of the work.

## Data availability

Qualified researchers may request data from Amgen clinical studies. Complete data are available at the following: https://wwwext.amgen.com/about/how-we-operate/policies-practices-and-disclosures/ethical-research/clinical-data-transparency-practices/clinical-trial-data-sharing-request.

## Declaration of competing interests

I Gouni-Berthold reports research grants from Amgen, Akcea, Novartis and Sanofi; speaker and consultancy fees from Amgen, Aegereon, Amarin, Akcea, Daiichi-Sankyo, Novartis, Pfizer, Regeneron and Sanofi. F Schaper reports no conflict of interest. U Schatz reports speaker and consultancy honoraria from Amgen, Daiichi-Sankyo, Diamed, Kaneka, Novartis, and Sanofi. A Tabbert-Zitzler, U Fraass and S Sauer are employees of Amgen and hold Amgen stock. K.K.R. reports research grants from Amgen, Regeneron, Daiichi Sankyo, Sanofi; consultancy fees from Novartis, Eli Lilly, Silence Therapeutics, New Amsterdam, Kowa, Esperion, Daiichi Sankyo, Cargene, Astra Zeneca, Bayer, Regeneron, Resverlogix, Roche, Abbott, New Amsterdam; and lecture fees from Novo Nordisk, Boehringer Ingelheim, Amgen, Viatris, Novartis, Sanofi, Daiichi Sankyo.

## References

[1] European Heart Network (2017).

[2] World Health Organisation (2021).

[3] (2020). Statistisches bundesamt deutschland.

[4] Giugliano R.P., Cannon C.P., Blazing M.A. (2018). Benefit of adding ezetimibe to statin therapy on cardiovascular outcomes and safety in patients with versus without diabetes mellitus: results from IMPROVE-IT (improved reduction of outcomes: vytorin efficacy international trial). Circulation.

[5] Sabatine M.S., Giugliano R.P., Keech A.C. (2017). Evolocumab and clinical outcomes in patients with cardiovascular disease. N Engl J Med.

[6] Schwartz G.G., Steg P.G., Szarek M. (2018). Alirocumab and cardiovascular outcomes after acute coronary syndrome. N Engl J Med.

[7] Weitgasser R., Ratzinger M., Hemetsberger M. (2018). [LDL-cholesterol and cardiovascular events: the lower the better?. Wien Med Wochenschr.

[8] Besseling J., van Capelleveen J., Kastelein J.J. (2013). LDL cholesterol goals in high-risk patients: how low do we go and how do we get there?. Drugs.

[9] Mach F., Baigent C., Catapano A.L. (2020). 2019 ESC/EAS Guidelines for the management of dyslipidaemias: lipid modification to reduce cardiovascular risk. Eur Heart J.

[10] Catapano A.L., Graham I., De Backer G. (2016). 2016 ESC/EAS guidelines for the management of dyslipidaemias. Eur Heart J.

[11] De Backer G., Jankowski P., Kotseva K. (2019). Management of dyslipidaemia in patients with coronary heart disease: results from the ESC-EORP EUROASPIRE V survey in 27 countries. Atherosclerosis.

[12] Kotseva K., De Backer G., De Bacquer D. (2019). Lifestyle and impact on cardiovascular risk factor control in coronary patients across 27 countries: results from the European Society of Cardiology ESC-EORP EUROASPIRE V registry. Eur J Prev Cardiol.

[13] Fox K.M., Tai M.-H., Kostev K. (2018). Treatment patterns and low-density lipoprotein cholesterol (LDL-C) goal attainment among patients receiving high- or moderate-intensity statins. Clin. Res. Cardiol.Off. J. Ger. Card. Soc..

[14] Gitt A.K., Lautsch D., Ferrieres J. (2017). Cholesterol target value attainment and lipid-lowering therapy in patients with stable or acute coronary heart disease: results from the Dyslipidemia International Study II. Atherosclerosis.

[15] Gitt A.K., Lautsch D., Ferrieres J. (2018). Contemporary data on treatment practices for low-density lipoprotein cholesterol in 6794 patients with stable coronary heart disease across the world. Data Brief.

[16] Schwaab B., Zeymer U., Jannowitz C. (2019). Improvement of low-density lipoprotein cholesterol target achievement rates through cardiac rehabilitation for patients after ST elevation myocardial infarction or non-ST elevation myocardial infarction in Germany: results of the PATIENT CARE registry. Eur J Prev Cardiol.

[17] Schmidt N., Dressel A., Grammer T.B. (2018). Lipid-modifying therapy and low-density lipoprotein cholesterol goal attainment in patients with familial hypercholesterolemia in Germany: the CaReHigh Registry. Atherosclerosis.

[18] Marz W., Dippel F.W., Theobald K. (2018). Utilization of lipid-modifying therapy and low-density lipoprotein cholesterol goal attainment in patients at high and very-high cardiovascular risk: real-world evidence from Germany. Atherosclerosis.

[19] Stock J.K., DA VINCI study (2020). Change in approach to cholesterol management will be needed to reduce the implementation gap between guidelines and clinical practice in Europe. Atherosclerosis.

[20] Ray K.K., Molemans B., Schoonen W.M. (2021). EU-wide cross-sectional observational study of lipid-modifying therapy use in secondary and primary care: the DA VINCI study. Eur J Prev Cardiol.

[21] Siostrzonek P., Brath H., Zweiker R. (2021).

[22] Vrablik M., Seifert B., Parkhomenko A. (2021). Lipid-lowering therapy use in primary and secondary care in Central and Eastern Europe: DA VINCI observational study. Atherosclerosis.

[23] Conroy R.M., Pyörälä K., Fitzgerald A.P. (2003). Estimation of ten-year risk of fatal cardiovascular disease in Europe: the SCORE project. Eur Heart J.

[24] Wilson P.W., D'Agostino R Sr., Bhatt D.L. (2012). An international model to predict recurrent cardiovascular disease. Am J Med.

[25] Gitt A.K., Junger C., Smolka W. (2010). Prevalence and overlap of different lipid abnormalities in statin-treated patients at high cardiovascular risk in clinical practice in Germany, Clinical research in cardiology. official journal of the German Cardiac Society.

[26] Gitt A.K., Rieber J., Hambrecht R. (2018). Do acute coronary events affect lipid management and cholesterol goal attainment in Germany? : results from the Dyslipidemia International study II. Wien Klin Wochenschr.

[27] Gitt A.K., Sonntag F., Jannowitz C. (2016). Better lipid target achievement for secondary prevention through disease management programs for diabetes mellitus and coronary heart disease in clinical practice in Germany. Curr Med Res Opin.

[28] Ludt S., Angelow A., Baum E. (2017). Deutsche Gesellschaft für Allgemeinmedizin und Familienmedizin (DEGAM).

[29] Bundesärztekammer (BÄK) (2019).

[30] Ray K.K., Reeskamp L.F., Laufs U. (2021). Combination lipid-lowering therapy as first-line strategy in very high-risk patients. Eur Heart J.

[31] Ray K.K. (2021). Changing the paradigm for post-MI cholesterol lowering from intensive statin monotherapy towards intensive lipid-lowering regimens and individualized care. Eur Heart J.

[32] Grundy S.M., Stone N.J. (2019). 2018 American heart association/American College of cardiology multisociety guideline on the management of blood cholesterol: primary prevention. JAMA Cardiol.

