# Supplementary material for: Low-density lipoprotein cholesterol goal attainment in Germany: Results from the DA VINCI study
Source: Atheroscler Plus. 2022 Aug 8;50:10–6. doi: 10.1016/j.athplu.2022.07.024 (PMC9833225; doi:10.1016/j.athplu.2022.07.024)
Supplement: Multimedia component 1 [file mmc1.docx]

**Supplementary Appendix**

**Low-density lipoprotein cholesterol goal attainment in Germany: Results from the DA VINCI Study**

**Authors**

**Ioanna Gouni-Berthold, Frank Schaper, Ulrike Schatz, Anja Tabbert-Zitzler, Uwe Fraass, Sarah Sauer, Kausik K. Ray**

**Figure S1. Study overview**


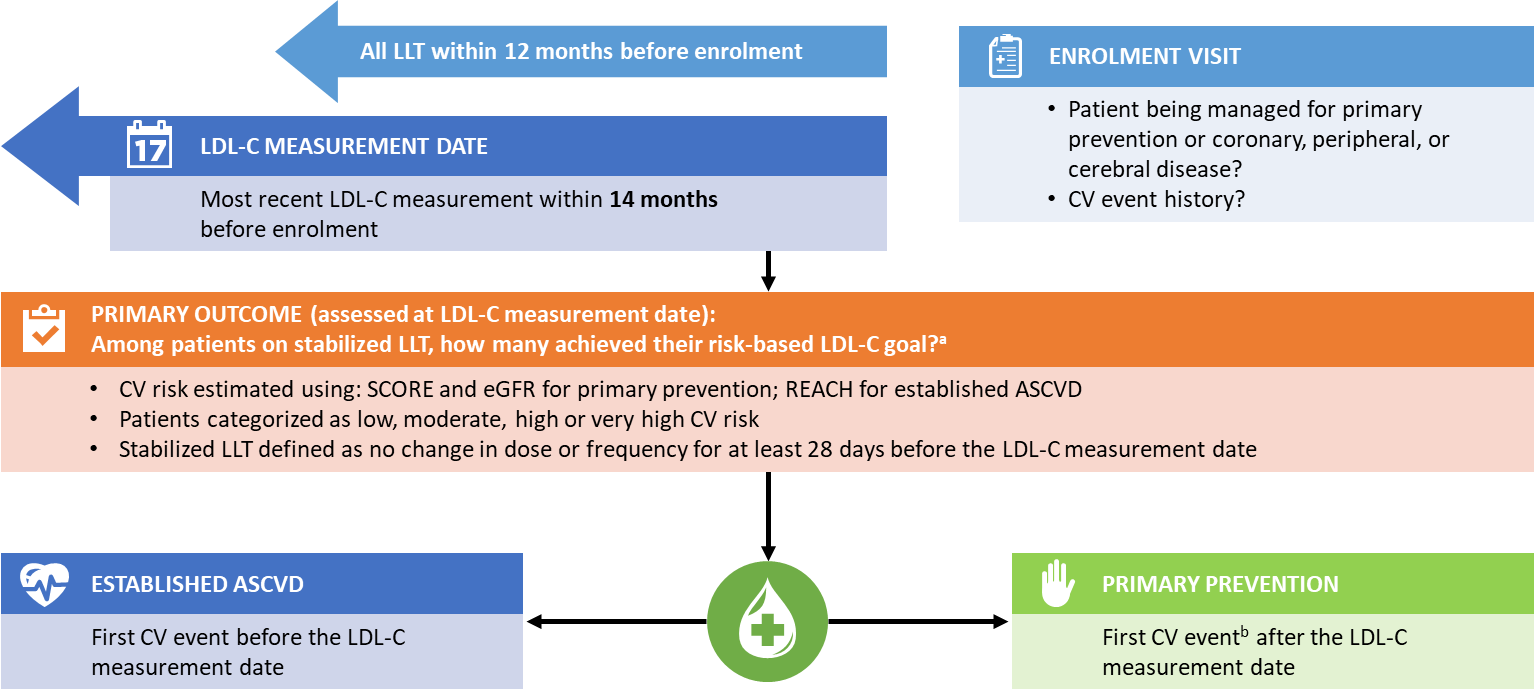


ASCVD = atherosclerotic cardiovascular disease; CV = cardiovascular; eGFR = estimated glomerular filtration rate; LDL-C = low-density lipoprotein cholesterol; LLT = lipid-lowering therapy; REACH = Reduction of Atherothrombosis for Continued Health; SCORE = Systematic Coronary Risk Evaluation.

Patients who were not stabilized on any LLT or had their LDL-C measurement taken before any LLT was initiated were not included in the assessment of the primary outcome. Includes patients enrolled as primary prevention with no CV events.

^a^ Patients enrolled as secondary prevention who were not being managed for peripheral, vascular or coronary disease and who had other evidence of atherosclerosis or other manifestations of vascular disease at enrolment. ^b^ Among patients considered as secondary prevention at the enrolment visit, 142 had their first CV event recorded after their most recent LDL-C measurement, and so were analysed as primary prevention patients for outcomes assessed at LDL-C measurement, such as goal attainment. For outcomes assessed at enrolment, these 142 patients were analysed as secondary prevention patients.

Reproduced from Ray KK, Molemans B, Schoonen WM, Giovas P, Bray S, Kiru G, et al. EU-Wide Cross-Sectional Observational Study of Lipid-Modifying Therapy Use in Secondary and Primary Care: the DA VINCI study. Eur J Prev Cardiol. 2021;28(11):1279-89 under a Creative Commons Attribution Non-Commercial License (<http://creativecommons.org/licenses/by-nc/4.0/>). © The authors 2020.

**Figure S2. Patient** **distribution by ASCVD status**

**
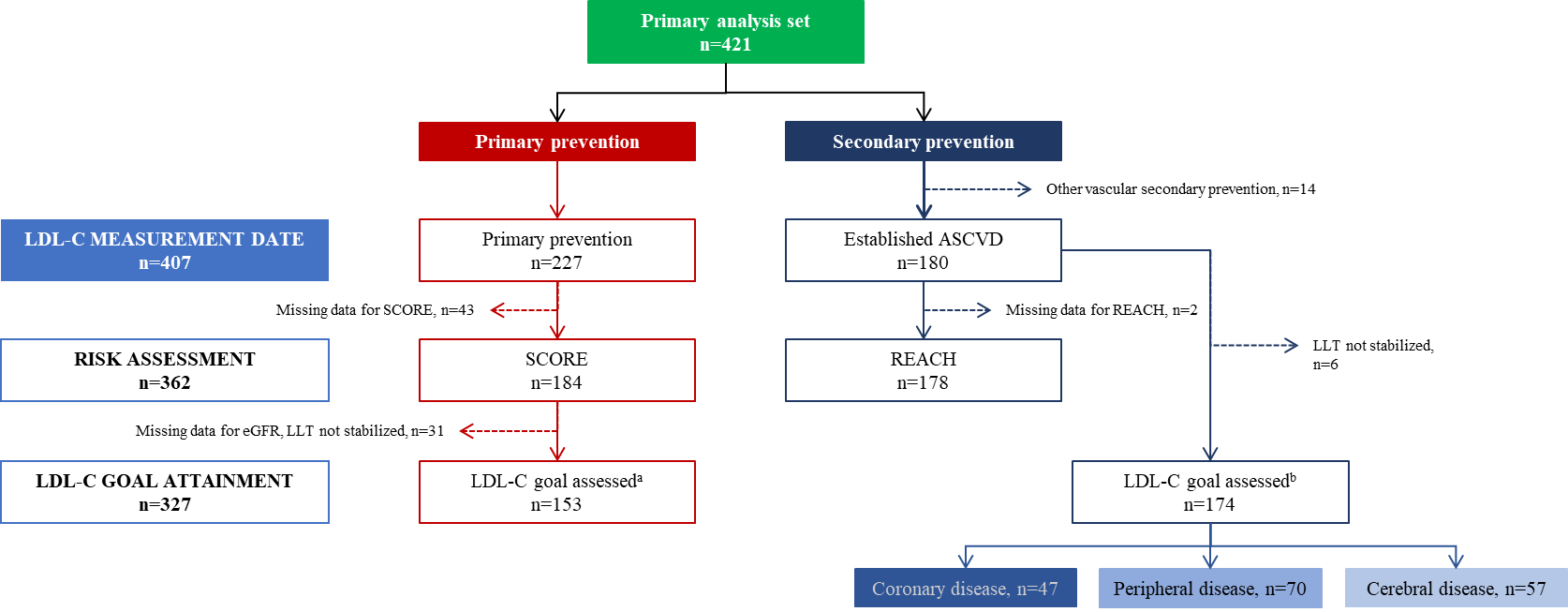
**

ASCVD = atherosclerotic cardiovascular disease; CV = cardiovascular; eGFR = estimated glomerular filtration rate; LDL-C = low-density lipoprotein cholesterol; LLT = lipid-lowering therapy; SCORE = Systematic Coronary Risk Evaluation.

^a^LDL-C goal attainment was assessed in primary prevention patients who received stabilized LLT and had available LDL-C measurement, SCORE assessment and eGFR measurement; ^b^LDL-C goal attainment was assessed in secondary prevention patients who received stabilized LLT and had available LDL-C measurement; all secondary prevention patients were considered very high-risk patients, irrespective of the REACH score.

ASCVD types were categorized by the investigator at enrolment as coronary, cerebrovascular or peripheral (mutually exclusive categories) based on the vascular bed primarily being managed at enrolment. However, some patients also had another vascular bed affected, which is captured in Table 1 of the publication.

**Figure S3. Cardiovascular risk scores**

**A. SCORE (primary prevention)^a^**


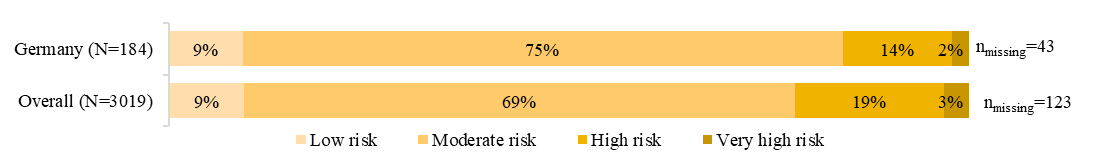


**B. REACH score (secondary prevention)^b^**


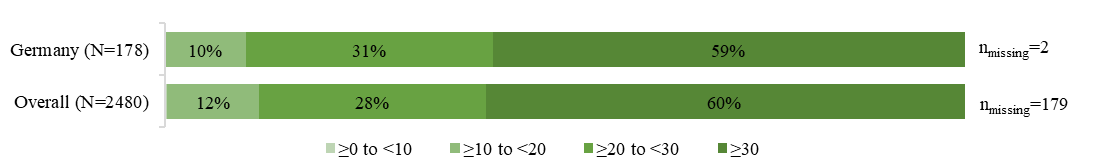


REACH = Reduction of Atherothrombosis for Continued Health; SCORE = Systematic Coronary Risk Evaluation

^a^Ten-year risk of CV death was calculated using the SCORE tool for 184 of 227 primary prevention patients from the German cohort and 3019 of 3142 from the overall study population. ^b^Ten-year risk of next CV event was calculated using REACH for 178 of 180 secondary prevention patients from the German cohort and 2480 of 2659 from the overall study population. For the remain patients, missing data prevented calculation of SCORE or REACH, respectively.

For more details on the overall study population please refer to Ray, KK, Molemans, B, Schoonen, WM, et al., EU-Wide Cross-Sectional Observational Study of Lipid-Modifying Therapy Use in Secondary and Primary Care: the DA VINCI study, Eur J Prev Cardiol, 2021;28:1279-1289.
